# Economic evaluation in healthcare implementation: a systematic review

**DOI:** 10.3389/frhs.2026.1838074

**Published:** 2026-10-21

**Authors:** Katarina Ulfsdotter Gunnarsson, Per Nilsen

**Affiliations:** Department of Health, Medicine and Caring Sciences, Division of Society and Health, Linköping University, Linköping, Sweden

**Keywords:** cost-effectiveness, economic evaluation, implementation science, implementation strategies, systematic review

## Abstract

**Background:**

Economic evaluations are critical for guiding resource allocation in healthcare but remain underutilized in implementation science. While implementation strategies such as training, infrastructure change and facilitation incur substantial costs, few studies assess their economic value. This systematic review synthesizes evidence on how economic evaluations have been applied to implementation efforts in healthcare since 2016.

**Methods:**

We conducted a systematic review following PRISMA guidelines, registered in PROSPERO (CRD420251018265). Searches were performed in PubMed, Scopus, PsycInfo, PsycArticles and trial registries for studies published after April 2016. Eligible studies included economic evaluations embedded in healthcare implementation efforts. Data extraction covered study context, implementation strategies, economic methods and quality assessed using the Drummond checklist.

**Results:**

From 5,729 screened records, 22 studies were included. Most originated from the United States, with others from Australia, Africa, Asia, Denmark, and one multicountry study. Training and education were the most common implementation strategies. Sixteen studies reported cost-only analyses, while six conducted full economic evaluations: three cost-effectiveness, one cost-utility analysis, one cost-benefit analysis, and one cost-minimization analysis. Standardized health outcome metrics were rarely used; only one study used DALYs and one study used life-years gained. Reporting of perspective, time horizon and cost categories was inconsistent, and implementation and intervention costs were not always clearly distinguished.

**Conclusion:**

Economic evaluation in implementation science remains limited, dominated by descriptive cost studies with major methodological gaps. Without linking costs to outcomes, evidence provides little policy guidance on value for money. More rigorous, prospective economic evaluations are needed, including standardized metrics and clear reporting, to ensure implementation strategies are scalable, sustainable and relevant to healthcare decision-makers.

**Systematic Review Registration:**

PROSPERO CRD420251018265.

## Contributions to the literature

- Synthesizes recent economic evaluations embedded in implementation science, highlighting progress since 2016.
- Identifies a continued dominance of cost-only studies (16 of 22), with only six full economic evaluations: three cost-effectiveness analyses, one cost-utility analysis, one cost-benefit analysis, and one cost-minimization analysis.
- Highlights major methodological gaps, such as lack of cost perspective, time horizon reporting and distinction between implementation and intervention costs.
- Demonstrates the limited use of standardized health outcome metrics such as QALYs and DALYs, restricting cross-study comparability.
- Emphasizes the need for prospective, rigorous economic evaluation design in implementation studies, especially for policy relevance and transferability.

## Introduction

Health systems around the world face persistent resource constraints, making the efficient allocation of funding and services a critical priority. In healthcare settings, where decisions must balance patient needs, clinical outcomes and limited organizational resources, economic evaluations such as cost-effectiveness, cost-utility and cost-benefit analyses have long served as foundational tools for assessing the value of evidence-based clinical interventions (1). However, these evaluations have traditionally concentrated on the clinical efficacy/effectiveness and economic efficiency of the interventions themselves, often overlooking the substantial resource demands associated with implementing those interventions in real-world settings (2). Implementation strategies within the healthcare setting, such as workforce training, infrastructure modifications and system-level adaptations, can significantly influence both the feasibility and sustainability of evidence-based interventions (3, 4).

Despite their relevance, economic evaluations remain relatively rare in implementation science (5). A 2019 review by Roberts et al. (6) highlighted the still developmental nature of economic inquiry within the field, pointing to substantial gaps in cost reporting and methodological rigor. Similarly, a 2022 scoping review by Malhotra et al. (7) emphasized the need for stronger methodological standardization in economic evaluation, particularly in low- and middle-income country (LMIC) settings. Crucially, without systematic assessment of resource use, it is difficult to discern whether reported implementation outcomes were achieved through scalable, pragmatic strategies or through resource-intensive supports such as dedicated staff, external facilitation or financial incentives that may not be replicable in typical care settings.

Both the interventions and the strategies used to implement them entail opportunity costs: investing in one approach may preclude investment in potentially more cost-effective alternatives (8). In health systems characterized by finite resources and growing demand, the absence of economic considerations in implementation science studies risks guiding policy and practice towards options that are effective in controlled settings but ultimately unsustainable or inefficient in routine healthcare environments (9).

In recent years, there has been increasing recognition within implementation science of the need to embed economic evaluations into empirical studies to more effectively inform healthcare decision-making (6, 10). This systematic review contributes to that effort by synthesizing economic evaluations conducted as part of implementation studies in healthcare settings since 2016. Specifically, the types of economic analyses used, the implementation strategies assessed and the methodological quality of the evaluations are examined. Thus, it provides a comprehensive overview of the current state of economic integration in implementation science and identifies key directions for future research and practice.

## Methods

We conducted a systematic review of research literature. We pre-registered the review protocol on the prospective register of systematic reviews (PROSPERO) (CRD420251018265). The review is reported according to the Preferred Reporting Items for Systematic reviews (PRISMA) checklist.

We searched PubMed, Scopus, PsycInfo and PsycARTICLES for peer-reviewed reports in the English language. Grey literature was excluded. The International Clinical Trials Registry Platform (WHO), the International Standard Randomized Controlled Trial Number and ClinicalTrials.gov trial registration databases were also searched. We developed a search strategy following the approach used by Roberts et al. (2019), to capture literature published after April 2016 to align with the endpoint of their prior review. The database search for the review was conducted on April 6, 2025, and an updated search was made on June 15, 2026.

The search strategy was developed around three core concepts: (1) implementation or implementation strategies (2) economic analyses, and (3) healthcare settings. For each concept we combined relevant keywords and controlled vocabulary terms (e.g., MeSH). See the Supplementary Information for the search query.

### Study inclusion and exclusion criteria

Eligibility criteria were structured using a PICO-informed framework, please see Table 1. The population of interest in this review was healthcare providers. The healthcare setting refers to, for example, hospitals, primary and community care clinics. We excluded studies conducted in non-healthcare settings such as schools or patients’ homes. This criterion ensures contextual relevance because implementing practices in environments such as primary care, hospitals, and specialist clinics presents unique challenges and opportunities.

**Table 1:** PICO and eligibility criteria.

Eligibility critera	Inclusion critera
Population	Healthcare providers delivering care within healthcare settings
Intervention	Implementation strategies aimed at promoting uptake of an evidence-based practice
Comparator	Usual care or alternative implementation strategy (where applicable)
Outcomes	Studies reporting economic outcomes (cost analysis or full/partial economic evaluation)
Study type and language	Peer-reviewed empirical studies published in English
Publication year	≥2016

To be included, studies had to involve evidence-based implementation objects (i.e., the specific clinical practice, intervention, programme or other entity being implemented), aimed at improving patient health to ensure that the review remained directly relevant to patient care and health outcomes, delivered by healthcare providers operating within healthcare settings.

Studies were included if they examined strategies (also referred to as methods, interventions or facilitation efforts) designed to promote the uptake of the implementation objects. Comparator conditions are the alternative strategy or standard practice that a study uses as a comparison to evaluate the effectiveness. This criterion ensures alignment with the core aims of implementation science by focusing the review on efforts to bridge the gap between research and practice, consistent with widely accepted definitions of implementation science as “the study of methods to promote the systematic uptake of research findings and other evidence-based practices into routine practice and, hence, to improve the quality and effectiveness of health services” (11).

Eligible studies were required to report economic outcomes, including cost analyses (cost-only studies) as well as full or partial economic evaluations such as cost-effectiveness, cost-utility analysis, cost-benefit, or cost-minimizing analysis.

### Screening and data extraction strategy

All titles and abstracts retrieved from the search were independently screened by one author (KUG) to identify potentially relevant studies (reasons for exclusion at this stage were not obtained). Independent screening by a second reviewer at the title and abstract stage was not feasible because the review was conducted without dedicated funding or additional review personnel. Full-text articles were obtained for studies that appeared to meet the eligibility criteria, and these were reviewed by both authors (KUG and PN). Any discrepancies were resolved through discussion. Key reasons for exclusion at the full-text stage were documented (Supplementary Information, Supplementary Table S1). Although dual independent screening at all stages is considered best practice, this approach was intended to reduce, but could not eliminate, the risk of selection error introduced by single-reviewer title and abstract screening.

We extracted data on study context and population, categories of implementation strategies, type of economic evaluation, study outcomes, cost categories and health economic results. We adhered to the terminology used in various studies. This means that different terms may refer to the same concept (synonymy), and the same term may represent different concepts (polysemy). Studies were synthesized narratively and grouped thematically rather than described individually. Themes were developed based on similarities in implementation strategies, economic evaluation methods, and study findings to facilitate comparison across the included evidence.

Implementation costs that were collected as a component of the economic evaluation were categorized using the Global Health Cost Consortium (GHCC) typology (Table 3), which categorizes costs: capital costs (e.g., office space/venues if owned), recurrent goods (e.g., rented office space/venues, training supplies, materials and utilites) personnel costs (e.g., supervision staff, project staff and technical assistance) and patient cost (e.g., travel costs).

### Study quality assessment

The methodological quality and risk of bias of the economic evaluation studies were assessed using the Drummond checklist (12). The Drummond checklist is specifically designed for assessing the quality of economic evaluations. This checklist consists of 35 items, each rated as ‘yes’, ‘no’, ‘not clear’, or, for certain methodological and analytical aspects, not appropriate’. Study quality was classified based on the proportion of applicable items rated “Yes”: high quality (>75%), moderate quality (50%–75%), and low quality (<50%). Other studies have used these rating classifications (13). Because several checklist domains were not applicable to cost-only analyses, we did not assign the 16 cost-only studies equivalent overall methodological-quality or risk-of-bias classifications. Instead, relevant checklist items were used descriptively to assess reporting characteristics, including analytical perspective, time horizon, cost categories, cost boundaries, and the identification, measurement, and valuation of resource use.

## Results

### Study selection

The database search yielded 5,729 records. After removal of duplicates (*n* = 691) and title/abstract screening (*n* = 5,038), full texts were assessed for eligibility. Twenty-two studies met the inclusion criteria. The main reasons for exclusion at full-text review were that studies were not conducted in healthcare settings, did not include an evidence-based practice, or healthcare workers were the intended recipients. The study selection process is presented in Figure 1 (PRISMA flowchart). See Table 2 for a summary of implementation characteristics and Table 3 for economic characteristics.

**Figure 1:**
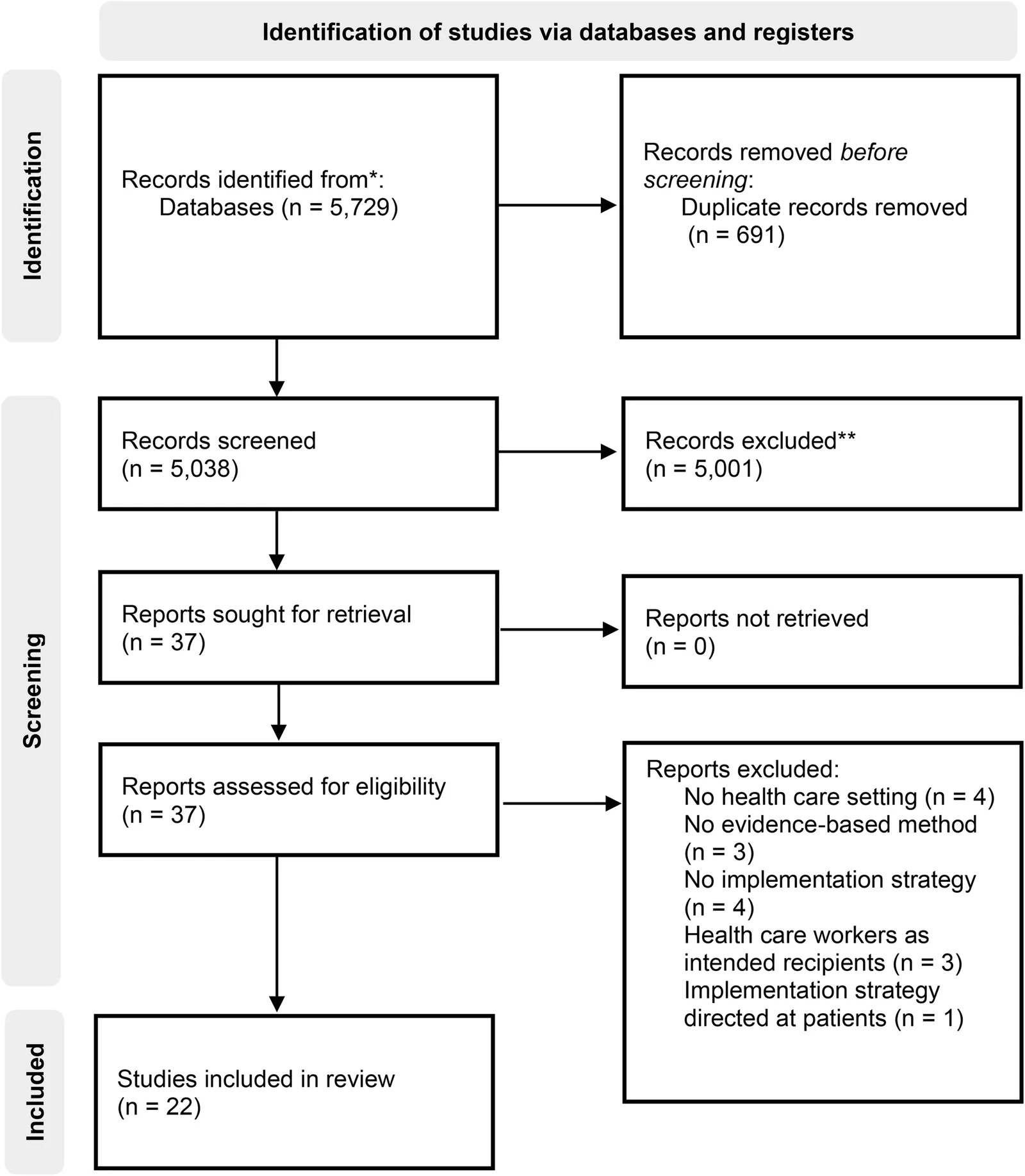
PRISMA flowchart.

**Table 2:** Implementation characteristics.

Author(s) (year)	Implemented practice (implementation object)	Evidence for the effectiveness of the implemented practice	Strategy or strategies used to support the implementation	Care setting for the implementation	Country where the study was conducted	Health issue being addressed	Healthcare providers who delivered the implemented practice	Intended recipients
Barbosa et al. (2018) (14)	Screening, Brief Intervention, and Referral to Treatment (SBIRT)	Evidence claimed but reported elsewhere	Technical assistance (support and feedback for practice managers and providers) and quarterly booster training for healthcare providers	Primary care and health centres	United States	Substance use	Primary care providers (Generalist model) and behavioral health specialists (Specialist model)	Adolescents
Barbosa et al. (2022) (15)	Screening, Brief Intervention, and Referral to Treatment (SBIRT)	Evidence claimed but reported elsewhere	Technical assistance (support and feedback for practice managers and providers) and quarterly booster training for healthcare providers	Primary care and health centres	United States	Substance use	Primary care providers (Generalist model) and behavioral health specialists (Specialist model)	Adolescents
Bartholomew et al. (2021) (16)	Syringe service programme	Evidence claimed but reported elsewhere	Students performing unpaid work	Wound care clinic	United States	Blood-borne infections	Healthcare providers	People who inject drugs
Blackburn et al. (2025) (33)	Group sessions focused on social support, stigma reduction, and self-efficacy in seeking healthcare. Clinic provides youth-friendly HIV testing and sexual and reproductive health services	Evidence not addressed	Training and education for peer navigators (adolescents from the population of focus) and their supervisors	Community-based clinics	Zambia	HIV	Peer navigators and healthcare providers	Adolescent girls and young women
Chavez (2023) (17)	Psychiatric consultation and navigation programme supporting safer antipsychotic use through decision tools, specialist consultations, and telemental health	Evidence claimed but reported elsewhere	Healthcare providers received guidelines and options to adjust treatment, consult a psychiatrist, or expedite mental health support	Psychiatry care	United States	Issues related to use of antipsychotics	Primary care and behavioral health providers	Children and adolescents aged 3–17 years prescribed antipsychotic medications
Christensen et al. (2026) (34)	Physician-led medication reviews for patients with severe mental disorder and diabetes.	Evidence (from RCT) reported	Interdisciplinary collaboration/dialogue; weekly interdisciplinary case conferences; clinical pharmacologist-led medication review with recommendations to treating clinicians.	Psychiatry care	Denmark	Severe mental disorder and diabetes	Healthcare providers	Psychiatric outpatients with severe mental disorder and diabetes.
Duan et al. (2021) (18)	Transradial approach (TRA) for cardiac catheterization/ percutaneous coronary intervention, replacing or increasing use relative to the transfemoral approach (TFA).	Evidence claimed but reported elsewhere	Peer coaching consisting of a training program and on-site coaching.	Hospital-based cardiovascular services	United States	Coronary heart disease requiring cardiac catheterization/ percutaneous coronary intervention.	Healthcare providers	Patients undergoing cardiac catheterization and/or percutaneous coronary intervention.
Hoeft et al. (2019) (19)	Collaborative care for treating depression	Evidence not addressed	Personnel support	Primary care	United States	Depression	Primary care providers	Older adults aged 65 + years
Jordan et al. (2019) (20)	Family-based intervention to prevent paediatric obesity	Evidence claimed but reported elsewhere	Developing automated fidelity coding for monitoring, developing and tailoring clinical materials and training coordinators	Primary care	United States	Paediatric obesity	Primary care providers	Families
Keenan-Devlin et al. (2024) (21)	Clinically-integrated breastfeeding peer counselling programme	Evidence (from RCT) reported	Multidisciplinary clinical support team developed workflows that integrated the breastfeeding peer counsellor into the clinic and the postpartum unit	Community health centre	United States	Breastfeeding	Peer navigators (adolescents from the population of focus) and healthcare providers	Mothers
Kurzawa et al. (2021) (31)	Iron and folic acid supplementation programme	Evidence (from RCT) reported	Training for healthcare providers	Antenatal care	Bangladesh	Anaemia among pregnant women	Healthcare providers	Pregnant women
Lane et al. (2021) (22)	Prevention programme to identify and address psychosocial risk factors for child maltreatment	Evidence claimed but reported elsewhere	Training, booster sessions and development and distribution of materials	Paediatric primary care	United States	Child maltreatment	Paediatric providers	Children and adolescents
Lang and Connell (2017) (23)	Trauma-focused cognitive behavioral therapy	Evidence claimed but reported elsewhere	Training, supervision, and implementation support	Outpatient children's behavioral health clinics, housed in community-based behavioral health agencies	United States	Mental health	Healthcare providers	Children
Nichols et al. (2020) (32)	HIV self-testing programme with provider-initiated counselling, facility-based kit use, and private interpretation with tailored support	Evidence not addressed	Training for healthcare providers	Outpatient care	Malawi	HIV	Healthcare providers	Adults
Oxlade et al. (2021) (35)	Identification, screening, evaluation, and initiation of tuberculosis preventive treatment	Evidence (from RCT) reported	A three-phase health systems strengthening intervention involving programme evaluation, stakeholder-led decision-making, and implementation of locally tailored improvement activities.	Tuberculosis clinics	Benin, Canada, Ghana, Indonesia, and Vietnam.	Tuberculosis	Healthcare providers	Household contacts of patients with microbiologically confirmed pulmonary tuberculosis.
Parkinson et al. (2026) (26)	Lynch syndrome genetic testing and genetics referral pathway for colorectal cancer patients	Evidence claimed but reported elsewhere	Theory-based implementation strategy compared with intuition-based implementation strategy	Hospital	Australia	Lynch syndrome	Healthcare providers	Patients undergoing colorectal cancer resection
Rahja et al. (2020) (27)	Reablement programme combining occupational therapy for functional outcomes with nursing for medical care	Evidence claimed but reported elsewhere	Training for occupational therapists	Community geriatric services	Australia	Dementia	Occupational therapists	People with dementia and their caregivers
Rose et al. (2025) (24)	Pediatric oral health intervention involving provider education, dental referrals, and electronic medical record based oral health assessments.	Evidence claimed but reported elsewhere	Electronic medical record enhancement by integrating oral health assessment questions; provider training; oral health educational materials/visual aids; dental visit prescription template; list of Medicaid-accepting dentists; initial training and later booster training.	Pediatric primary care practices	United States	Dental caries and untreated dental decay	Primary care providers	Adult caregivers and their 3- to 6-year-old Medicaid-enrolled children.
Wagner et al. (2021) (29)	Safer Conception Counselling programme supports informed reproductive decisions with training in contraception or safer conception methods	Evidence (from RCT) reported	Two training strategies were tested: high- or low-intensity strategy, differentiated by amount of training and supervision	HIV clinics	Uganda	HIV	HIV counsellors	HIV-serodiscordant couples
Willcox et al. (2019) (30)	Low-dose, high-frequency training for competency, team-based repetitive learning and practice	Evidence claimed but reported elsewhere	Training for healthcare providers	Health centres and hospitals	Uganda	Post-abortion care and paediatric HIV	Healthcare providers	Women
Woolf et al. (2024) (28)	Group-based cognitive training programme	Evidence (from RCT) reported	Education and training for healthcare providers	Mental healthcare	Australia	Major depressive disorder	Healthcare providers	Adults ≥65 years
Yeung et al. (2020) (25)	Behavior health integration programme involving population-based screening assessment and identification	Evidence claimed but reported elsewhere	Three strategies for healthcare providers: (1) practice coaching; (2) electronic health records clinical decision support; and (3) performance monitoring and feedback	Primary care	United States	Depression, suicidal thoughts and/or alcohol or substance use disorders	Primary care providers	Primary care patients

The term “healthcare providers” is used generically in the tables to refer to clinicians, healthcare workers and those in other similar roles. However, when studies used more specific terminology, such as paediatric providers, social workers or HIV counsellors, those terms are used accordingly. RCT, randomized controlled trial.

**Table 3:** Economic characteristics.

Author (year)	Type of economic evaluation	Perspective of the analysis	Implementation cost category or categories	Time horizon: measurements points	Study outcomes	Conclusions about cost-effectiveness
Barbosa et al. (2018) (14)	Cost-only study	-	Recurrent goods and personnel costs.	Start-up phase (6 months)	Total cost for the Generalist model (delivery by primary care providers) and Specialist model (delivery by behavioral health specialists)	–
Barbosa et al. (2022) (15)	Cost-effectiveness analysis	Healthcare	Recurrent goods and personnel costs.	1 and 5 years	Cost per additional positive screening (ICER)	Did not calculate ICER. The Generalist model is less expensive and more effective than the Specialist model
Bartholomew et al. (2021) (16)	Cost-only study	-	Recurrent goods and personnel costs.	Start-up phase and 1 year	Total cost of implementation and first year	–
Blackburn et al. (2025) (33)	Cost-only study	-	Recurrent goods and personnel costs.	July 2021 to December 2022	Total cost	–
Chavez et al. (2023) (17)	Cost-only study	-	Recurrent goods and personnel costs.	Start-up phase and 1 year	Total start-up and ongoing cost	–
Christensen et al. (2026) (34)	Cost-minimization analysis	Healthcare	Recurrent goods and personnel costs.	7 months	Total cost	The intervention was cost-neutral.
Duan et al. (2021) (18)	Cost-only study	Healthcare system	Recurrent goods and personnel costs.	March 2018—March 2020	Total cost	-
Hoeft et al. (2019) (19)	Cost-only study	-	Recurrent goods and personnel cost.	1 month	Total cost	–
Jordan et al. (2019) (20)	Cost-only study	-	Recurrent goods and personnel cost.	28 weeks	Total cost	–
Keenan-Devlin et al. (2024) (21)	Cost-only study	-	Recurrent goods and personnel cost.	1 year	Total cost	–
Kurzawa et al. (2021) (31)	Cost-utility analysis	Healthcare	Recurrent goods and personnel cost.	Not reported	Cost per DALY averted (ICER)	Intervention could be considered cost-effective compared to standard care.
Lane et al. (2021) (22)	Cost-only study	-	Recurrent goods and personnel cost.	1, 2 and 3 years	Total cost	–
Lang and Connell (2017) (23)	Cost-only study	-	Recurrent goods and personnel cost.	3 and 7 months	Incremental cost	–
Nichols et al. (2020) (32)	Cost-only study	-	Recurrent goods and personnel cost.	Not reported	Total cost, cost per person diagnosed	–
Oxlade et al. (2021) (35)	Cost-effectiveness analysis	Healthcare	Recurrent goods and personnel costs.	Approximately 20-month study period	Cost per additional contact initiating treatment	Intervention is cost-effective compared to standard care.
Parkinson et al. (2026) (26)	Cost-effectiveness analysis: trial-based cost analysis plus modelled economic evaluation	Healthcare	Recurrent goods and personnel costs.	5 years/lifetime	Cost per additional appropriately managed patient; life years gained (ICER)	The theory-based approach was more effective and less costly. In the modelled analysis, it produced more life-years gained and could be considered cost-effective
Rahja et al. (2020) (27)	Cost-benefit analysis	Market, private, efficiency (social) and referent group (key stakeholders).	Recurrent goods and personnel costs.	5 years	Costs and benefits (in monetary terms)	Benefits outweigh costs (societal gain).
Rose et al. (2025) (24)	Cost-only study	-	Recurrent goods and personnel costs.	First year implementation and subsequent annual maintenance cost	Total cost	-
Wagner et al. (2021) (29)	Cost-only study	-	Recurrent goods and personnel costs.	12 months	Cost per intensity approach	–
Willcox et al. (2019) (30)	Cost-only study	-	Recurrent goods and personnel cost.	9 months	Total cost	–
Woolf et al. (2024) (28)	Cost-only study	-	Recurrent goods and personnel cost.	Not reported	Total cost	–
Yeung et al. (2020) (25)	Cost-only study	-	Recurrent goods and personnel cost.	41 months	Total cost	–

DALY, disability-adjusted life year; ICER, incremental cost-effectiveness ratio.

### Study characteristics

The 22 studies were conducted in diverse countries and healthcare settings. Most were from the United States (*n* = 12) (14–25), followed by Australia (*n* = 3) (26–28) and Uganda (*n* = 2) (29, 30). Additional studies were conducted in Bangladesh (31), Malawi (32), Zambia (33) and Denmark (34), while one multicountry study included sites in Benin, Canada, Ghana, Indonesia and Vietnam (35).

### Types of implementation strategies

Training and education were the most frequently reported implementation strategies (15, 18, 22, 24, 27, 29–31, 33). Nearly all studies included some form of workforce training, either as a standalone strategy or combined with additional supports such as supervision, booster sessions, technical assistance or workflow redesign (14, 15, 18, 19, 22–24).

Other commonly used strategies included:

- Technical assistance and practice coaching (14, 25)
- Clinical decision support embedded in electronic health records (17, 24, 25)
- Performance monitoring and feedback (14, 25, 35)
- Development of new clinical workflows (19–21, 26, 34)
- Peer navigation and community engagement (21, 33)
- Supervision and fidelity monitoring (22, 23, 29)

In several studies, implementation involved multi-component strategies targeting organizational change, such as modifying care pathways, integrating new staff roles, embedding digital tools into existing systems, or combining training with coaching and locally selected improvement activities (17–19, 24–26, 34, 35).

However, many studies did not clearly distinguish between costs related to the implementation strategy (e.g., training time, technical assistance) and those related to delivery of the intervention itself (e.g., staff time for patient care) (14, 16, 22, 23). This lack of differentiation complicated interpretation of economic findings.

Across studies, implementation strategies varied not only in type but also in resource intensity. Brief educational or electronic-record-based strategies generally required fewer personnel and material inputs, whereas coaching, facilitation, supervision, and multicomponent health-system strengthening approaches required greater staff time, coordination, and ongoing support. This variation is important because studies that described strategies only as “training” often obscured differences in duration, intensity, delivery mode, and costs, limiting comparability across settings.

### Types of economic evaluations

#### Full economic evaluations

Six studies conducted economic evaluations (15, 26, 27, 31, 34, 35). These included:

- Three cost-effectiveness (15, 26, 35)
- One cost-utility analysis (31)
- One cost-benefit analysis (27)
- One cost-minimization analysis (34)

#### Cost-effectiveness and cost-utility analyses

Four studies calculated incremental cost-effectiveness ratios (ICERs) (15, 26, 31, 35). One used disability-adjusted life years (DALYs) as the outcome metric (31), one used life years gained (26), and two measured cost per implementation effectiveness outcome [e.g., additional positive screenings (15) and additional initiating of treatment (35)]. In two cases, the intervention model was both less costly and more effective, precluding calculation of an ICER (15, 26).

One of the cost-effectiveness studies used trial results to inform two existing microsimulation models, estimating the long-term health and economic consequences of improved implementation of Lynch syndrome testing and referral pathways. The models simulated disease incidence, life-years gained, and healthcare costs over a lifetime horizon (26).

Standardized outcome metrics such as Quality-adjusted life years (QALYs) and DALYs were not commonly employed in the included studies. DALYs were used in only one study (31) while one additional study used life-years gained (26). The limited use of standardized health outcome measures restricts comparability across studies and settings.

#### Cost-benefit analysis

One study conducted a cost-benefit analysis from multiple perspectives (market, private, societal and stakeholder) (27). Benefits were monetized and compared directly to costs over a five-year time horizon. The intervention demonstrated net societal gain, with benefits outweighing costs (27).

#### Cost-minimization analysis

One study conducted a cost-minimization analysis because the effect on clinical outcomes and quality of life of the intervention was equivalent between groups (34). The analysis was from a healthcare perspective, and cost was measured over an average of seven months. The intervention was cost-neutral, with no significant difference in total costs, while reducing prescribed medications, potentially inappropriate prescriptions, and telephone contacts (34).

#### Cost-only studies

Sixteen of the twenty-two included studies reported cost-only analyses (14, 16–25, 28–30, 32, 33). These studies typically estimated:

- Start-up costs (14, 16, 17)
- Ongoing operational costs (16, 19, 21, 24, 25)
- Personnel time costs (16–23, 25, 30, 33)
- Material and administrative expenses (16, 19, 22)

Most cost-only studies did not formally relate costs to effectiveness or health outcomes. Instead, they provided descriptive estimates of the resources required to implement the practice within a given healthcare setting (14, 16–25, 28–30, 32, 33). Two of the studies provide particularly concrete examples. Duan et al. (18) used activity-based costing and estimated a total implementation cost of US$374,863 for a peer-coaching strategy across seven Veterans Affairs medical centres, with travel and personnel time accounting for most costs. Rose et al. (24) estimated comparatively modest first-year implementation costs for a paediatric oral-health intervention—approximately US$580 to US$1,482 depending on practice size—with lower subsequent annual maintenance costs.

Personnel costs were consistently a major cost category across studies (16–25, 29, 32). These included staff time for training, supervision, programme delivery, workflow development, coordination and coaching. Personnel costs were primarily estimated using activity-based costing or micro-costing approaches, in which staff time devoted to implementation activities was measured through interviews, questionnaires, staff diaries, activity logs, observations, time-and-motion studies, or project records and then valued using role-specific salary or wage rates (14–18, 24, 26, 33–35). Other studies used prospective time reporting systems, structured cost surveys, or self-reported estimates of time spent on implementation activities (20, 23, 25, 29), while some relied on staffing allocations, programme expenditure data, or assumed time inputs rather than direct measurement of staff time (21, 22, 27, 28, 31, 32).

Research-related costs, such as evaluation and data collection expenses, were frequently excluded or not explicitly reported (16, 20, 22).

The full economic evaluations provided greater decision relevance than cost-only studies because they linked resource use to effectiveness, health outcomes, or monetized benefits. In contrast, cost-only studies were useful for budget planning but could not determine whether implementation strategies represented good value for money. This distinction was particularly important where personnel costs were high: without corresponding outcome data, higher implementation costs could not be interpreted as either inefficient or justified by greater effectiveness.

### Economic perspectives and methodological reporting

Reporting of key economic parameters varied substantially across studies.

#### Perspective

Only a minority of studies explicitly stated the analytical perspective (e.g., healthcare system, societal or stakeholder) (15, 18, 26, 33, 34). Most cost-only studies did not clearly define a perspective, limiting interpretability for decision-makers.

#### Time horizon

Time horizons were inconsistently reported. Some studies assessed short-term start-up costs [e.g., six months (14)], others reported annual operating costs (16, 21, 24), and a few extended analyses to multiple years (15, 22, 27). In several cases, the time horizon was unclear or not reported, e.g. (28, 31, 32),. Only one study used modelling techniques and extrapolated data beyond the study period, using a lifetime time horizon (26).

#### Cost categories

Using the GHCC typology, most reported costs fell into recurrent goods and personnel costs (14–25, 27–35).

Capital costs and patient costs were rarely detailed (14, 16, 22). Implementation and intervention costs were often aggregated, with limited clarity regarding boundaries between categories (14, 16, 22).

#### Geographic and contextual patterns

Although most studies were conducted in high-income countries, several studies from LMIC settings demonstrated the feasibility of incorporating economic analysis into implementation research. The multicountry ACT4 study by Oxlade et al. (35) was particularly informative because it prospectively assessed implementation and health-system costs alongside a cluster-randomized trial in Benin, Ghana, Indonesia and Vietnam as well as Canada. Other examples came from Bangladesh, Uganda, Malawi and Zambia (29–33).

However, the overall distribution remains skewed toward high-income contexts, particularly the United States (14–25), indicating limited global representation in economic implementation research.

### Study quality assessment

We assessed all included studies using the Drummond checklist to provide a consistent appraisal of economic-method reporting. Because only six studies were full economic evaluations, several checklist items were not applicable to the 16 cost-only studies, particularly items concerning comparative effectiveness and the valuation of consequences. The limited reporting of effectiveness data in cost-only studies should therefore not in itself be interpreted as a methodological flaw. The 16 cost-only studies were not assigned equivalent overall quality or risk-of-bias classifications. Their appraisal focused descriptively on reporting characteristics relevant to cost analysis. Across the review, the appraisal nevertheless highlighted recurring reporting limitations concerning analytical perspective, time horizon, cost boundaries and the distinction between implementation and intervention costs. Among the six full economic evaluations, three were classified as high quality because more than 75% of applicable Drummond checklist items were rated “Yes”: Parkinson et al. (26) 86%, Oxlade et al. (35) 80%, and Barbosa et al. (15) 77%. The remaining three were classified as moderate quality: Kurzawa et al. (31) 74%, Rahja et al. (27) 71%, and Christensen et al. (34) 60%. No full economic evaluation was classified as low quality. The Supplementary Information (Table S2) provides a detailed appraisal of each study.

## Discussion

This systematic review synthesizes recent efforts to incorporate economic evaluation into implementation science. The updated review includes 22 studies and confirms a strong predominance of cost-only analyses (16 of 22), with only six full economic evaluations that formally relate costs to outcomes through cost-effectiveness, cost-utility, cost-benefit or cost-minimization approaches, and only one of the six studies modelled outcomes beyond the study period.

The studies included also differed in their usefulness for decision-makers. Studies that prospectively collected cost and outcome data, stated a clear perspective, and reported a defined time horizon were more transferable and policy-relevant. By contrast, studies that aggregated implementation and intervention costs or omitted the analytical perspective were less able to inform decisions about scale-up or sustainability. Thus, the main limitation of the evidence base is not simply the small number of full economic evaluations, but also the inconsistent specification of the decision problem that each evaluation was intended to inform.

Our findings are consistent with, but also extend, previous reviews of economic evaluation in implementation science. Roberts et al.'s (6) reviewed studies published before 2016 and concluded that economic evaluation was still uncommon in implementation and improvement science, with many studies reporting costs descriptively rather than relating costs to outcomes. By focusing on studies published from 2016 onward, our review provides an updated assessment of whether this pattern has changed. The continued predominance of cost-only analyses in our review suggests that progress has been limited: implementation costs are increasingly being documented, but they are still rarely integrated into comparative economic evaluations capable of informing resource-allocation decisions.

Our review also differs methodologically from Carrandi et al. (10), who focused specifically on economic evaluations conducted alongside randomised implementation trials. In contrast, we applied broader eligibility criteria and included both full economic evaluations and cost-only studies embedded in healthcare implementation efforts. This broader scope allowed us to assess not only the methodological quality of formal economic evaluations, but also how implementation costs are identified, measured, categorized, and reported more generally across implementation science. The findings indicate that cost reporting itself remains heterogeneous, particularly regarding analytical perspective, time horizon, cost boundaries, and the separation of implementation costs from intervention-delivery costs.

The findings also align with Malhotra et al. (7), who highlighted the need for greater methodological standardization and more economic evaluation of implementation outcomes in LMICs. Although our review included examples from Bangladesh, Malawi, Uganda, Zambia, and a multicountry study including Benin, Ghana, Indonesia, and Vietnam, the evidence base remained dominated by studies from high-income countries, especially the United States. This pattern suggests that the limited global representation noted in previous reviews persists and that further investment is needed to develop economic implementation research that is transferable across diverse health-system contexts.

Only two economic evaluations used health outcome metrics (DALYs and life-years gained). Although QALYs and DALYs are not appropriate in every implementation context, their limited use reduces comparability and makes it more difficult to prioritise among competing implementation strategies across healthcare settings. At the same time, some studies illustrate that implementation-relevant outcomes can also be meaningful economic endpoints. Oxlade et al. (35), for example, estimated cost per additional household contact initiating tuberculosis preventive treatment, while Christensen et al. (34) used cost-minimization because clinical outcomes and quality of life did not differ between groups. These examples underline the need to match the economic method and outcome metric to the decision problem rather than apply a single metric mechanically.

Training was the most frequently reported implementation strategy, but the additional studies demonstrate substantial heterogeneity in what ‘training’ entails and in the resources required. Duan et al. (18) evaluated an expertise-intensive peer-coaching model in which travel and personnel opportunity costs were major cost drivers, whereas Rose et al. (24) estimated low costs for brief provider training combined with an electronic medical record modification. Oxlade et al. (35) embedded training within a broader, locally tailored health-systems strengthening process, and Christensen et al. (34) relied on interdisciplinary dialogue and specialist medication review. This variation reinforces the importance of specifying the active implementation components and separating their costs from the costs of delivering the clinical intervention itself. Key economic parameters such as time horizons and perspectives were also often omitted, which limits transferability across settings.

Three studies calculated cost-effectiveness to compare implementation strategies. Barbosa et al. (15), Kurzawa et al. (31) and Oxlade et al. (35) provided comparative evidence indicating that a Generalist SBIRT model, an iron and folic acid supplementation programme, and a tuberculosis health-systems strengthening intervention, respectively, offered favourable economic value relative to their comparators. Oxlade et al. (35) estimated the combined intervention and LTBI clinical-care cost at CA$1,348 per additional household contact initiating preventive treatment. In addition, Rahja et al. (27) reported a positive societal return in a cost-benefit analysis, while Christensen et al. (34) found physician-led medication review to be cost-neutral over the period assessed. Together, these studies illustrate the greater decision value generated when implementation costs are explicitly linked to outcomes or benefits.

Most studies originated from high-income countries, particularly the United States (*n* = 12) (14–25), reflecting a persistent imbalance in the implementation science literature. Nevertheless, the inclusion of Oxlade et al. (35) strengthens the international scope of the evidence because the same health-systems intervention was evaluated across Canada and four LMICs. The broader pattern remains consistent with recent syntheses showing substantial heterogeneity in how implementation costs are defined and measured and limited representation from LMIC settings (7).

Promising examples from Bangladesh, Malawi, Zambia, Uganda and the multicountry ACT4 study demonstrate the feasibility and relevance of economic evaluation in LMICs in Asia and Africa. Oxlade et al. (35) is especially notable for prospectively collecting resource-use and cost data alongside implementation and effectiveness outcomes across heterogeneous health systems. Greater investment in economic implementation science research in LMICs remains essential not only for equity and scalability but also for developing methods that capture the true costs and consequences of implementation across diverse settings.

The findings of this review reinforce the call for a paradigm shift in how implementation strategies are evaluated. Economic evaluations must be designed in parallel with implementation studies, not appended *post hoc*, to ensure that appropriate data on costs and effects are collected prospectively. Adherence to established economic guidelines (e.g., the Drummond checklist) should become standard practice, and funders should require explicit justification if economic analyses are omitted in implementation science research. Furthermore, future studies should consistently specify cost perspectives (e.g., health system or societal), include opportunity costs and transparently report data sources and assumptions.

Another important issue concerns the translation of economic evidence into decision-making processes. Even when cost and outcome data are available, the extent to which healthcare organizations and policy-makers draw on such information to guide implementation choices is not always evident. Institutional priorities, political considerations and contextual factors, such as workforce availability or patient preferences, may also play an important role in shaping real-world decisions, alongside economic evidence. Future research should therefore not only strengthen methodological rigour but also investigate the mechanisms by which economic evaluations can be effectively communicated and integrated into policy and organizational decision-making. This dual focus on methodological improvement and practical uptake would enhance the utility of economic evidence in advancing sustainable implementation strategies.

Several limitations should be considered when interpreting the findings of this review. First, title and abstract screening was conducted by a single reviewer because the review was undertaken without dedicated funding or additional review personnel. This is an important methodological limitation, as single-reviewer screening may increase the risk of selection bias and may have led to the inadvertent exclusion of relevant studies. Although full-text articles were reviewed by both authors and eligibility criteria were applied systematically, dual full-text review cannot fully eliminate the possibility that eligible studies were missed at the title and abstract stage. The findings should therefore be interpreted with this limitation in mind. Dual independent screening at all stages would have strengthened the methodological rigor of the review. In addition, the review was restricted to studies conducted in healthcare settings and published in English. While this decision was made to enhance contextual relevance and comparability, it may have excluded informative implementation studies conducted in community, social care, or other sectors, as well as non-English publications, thereby limiting generalizability.

Second, despite a comprehensive search across multiple databases and trial registries, it is possible that relevant studies were missed, particularly given the variability in terminology used to describe both implementation strategies and economic evaluations. Furthermore, the conclusions of this review are constrained by the quality and completeness of reporting in the included studies. Many primary studies provided limited detail on cost perspectives, time horizons, cost categories, and the distinction between implementation and intervention costs. This heterogeneity and incomplete reporting limited comparability across studies and precluded quantitative synthesis. Consequently, the findings should be interpreted as reflecting the current state of reporting and methodological practice in the field rather than definitive evidence regarding the economic value of implementation strategies.

## Conclusion

Despite encouraging examples of good practice, our review shows that the current economic evidence base in implementation science remains uneven, with most studies focused on documenting the resources required to implement evidence-based practices rather than conducting full comparative economic evaluations. Only by integrating rigorous, comparative economic analyses into implementation science research can we generate actionable evidence on what works, for whom, at what cost and in which settings. Without this progression, implementation science risks producing evidence that is effective in theory but unaffordable in practice.
