# Supplementary material for: Economic evaluation in healthcare implementation: a systematic review
Source: Front Health Serv. 2026 Oct 21;6:1838074. doi: 10.3389/frhs.2026.1838074 (PMC13733396; doi:10.3389/frhs.2026.1838074)
Supplement: Supplementary file 2 [file Table1.docx]

Economic Evaluation in Healthcare Implementation: A Systematic Review

# Supplementary information

## The search queries

### PsycInfo

(TI “implement*” OR AB “implement*” OR KW “implement*” OR TI “implement* science*” OR AB “implement* science*” OR KW “implement* science*” OR TI “Implement* Strat*” OR AB “Implement* Strat*” OR KW “Implement* Strat*”)

AND

(TI “cost*” OR AB “cost*” OR KW “cost*” OR TI “cost-benefit” OR AB “cost-benefit” OR KW “cost-benefit” OR TI “cost-effectiveness” OR AB “cost-effectiveness” OR KW “cost-effectiveness” OR TI “cost-effective*” OR AB “cost-effective *” OR KW “cost-effective*” OR TI “economic evaluation*” OR AB “economic evaluation*” OR KW “economic evaluation*” OR TI “cost-utility*” OR AB “cost-utility*” OR KW “cost-utility*” OR TI “health economic evaluation*” OR AB “health economic evaluation*” OR KW “health economic evaluation*” OR TI “cost analysis*” OR AB “cost analysis*” OR KW “cost analysis*” OR TI “economic impact*” OR AB “economic impact*” OR KW “economic impact*”)

AND

(TI “healthcare *” OR AB “healthcare *” OR KW “healthcare *” OR TI “health care” OR AB “health care” OR KW “health care” OR TI “hospital” OR AB “hospital” OR KW “hospital” OR TI “medical *” OR AB “medical*” OR KW “medical*” OR TI “clinical*” OR AB “clinical*” OR KW “clinical*” OR TI “health services*” OR AB “health services*” OR KW “health services*” OR TI “health system*” OR AB “health system*” OR KW “health system*” OR TI “patient care*” OR AB “patient care*” OR KW “patient care*”)

### PubMed

(((( "implement*"[Title/Abstract] OR "implement* science"[Title/Abstract] OR "implement* strat*"[Title/Abstract] OR "implement* framework"[Title/Abstract] OR "implement* research"[Title/Abstract] OR "implement* outcomes"[Title/Abstract] )) AND ("cost"[Title/Abstract] OR "cost-effectiveness analysis"[Title/Abstract] OR "cost-effectiveness"[Title/Abstract] OR "cost-effective"[Title/Abstract] OR "economic evaluation"[Title/Abstract] OR "cost-utility analysis"[Title/Abstract] OR "budget impact analysis"[Title/Abstract] OR "health economic evaluation"[Title/Abstract] OR "cost analysis"[Title/Abstract] OR "Health Care Costs"[MeSH Terms] OR "Cost-Benefit Analysis"[MeSH Terms] OR "Health Expenditures"[MeSH Terms])) AND ("healthcare intervention"[Title/Abstract] OR "health intervention"[Title/Abstract] OR "medical intervention"[Title/Abstract] OR "clinical intervention"[Title/Abstract] OR "hospital intervention"[Title/Abstract] OR "health services intervention"[Title/Abstract] OR "patient care intervention"[Title/Abstract] OR "Program Evaluation"[MeSH Terms] OR "Health Program Planning"[MeSH Terms])) AND ("hospital-based"[Title/Abstract] OR "primary care"[Title/Abstract] Title/Abstract] OR "patient-centered care"[Title/Abstract] OR "healthcare delivery"[Title/Abstract] OR "Hospitals"[MeSH Terms] OR "Health Services"[MeSH Terms] OR "Health Systems"[MeSH Terms] OR "Health Care"[MeSH Terms]") AND (2016/01/01:2025/12/31[Date - Publication])

### Scopus

TITLE-ABS-KEY ( "implement*" OR "implementation science" OR "implementation strategy*" OR "implementation framework" OR "implementation research" OR "implementation outcomes" ) AND TITLE-ABS-KEY ( "cost" OR "cost-effectiveness analysis" OR "cost-effectiveness" OR "cost-effective" OR "economic evaluation" OR "cost-utility analysis" OR "budget impact analysis" OR "health economic evaluation" OR "cost analysis" OR "health care costs" OR "cost-benefit analysis" OR "health expenditures" ) AND TITLE-ABS-KEY ( "healthcare intervention" OR "health intervention" OR "medical intervention" OR "clinical intervention" OR "hospital intervention" OR "public health intervention" OR "health services intervention" OR "patient care intervention" OR "program evaluation" OR "health program planning" ) AND TITLE-ABS-KEY ( "hospital-based" OR "primary care" OR "patient-centered care" OR "healthcare delivery" OR "health services" OR "health systems" OR "health care" OR "public health" ) AND PUBYEAR > 2016 AND PUBYEAR < 2025

## Table S1. Records that were excluded at the full-text review stage and the reasons for their exclusion.

| **Records excluded** | **Comment on exclusion** |
| --- | --- |
| Alonso, S., Cambaco, O., Maússe, Y., et al. Costs associated with delivering HPV vaccination in the context of the first year demonstration programme in southern Mozambique. BMC Public Health. 2019;19:1031. <https://doi.org/10.1186/s12889-019-7338-4> | Not conducted in a health care setting. |
| Brouwers J, Seys D, Claessens F, Wilder VA, Luk Bruyneel L, Ridder D, et al. The cost of a first and second hospital-wide accreditation in Flanders, Belgium, Int J Qual Health Care. 2022;34(3):mzac062. <https://doi.org/10.1093/intqhc/mzac062> | Not an exclusive health focus. Hospital accreditation cannot be considered an evidence-based (or empirically grounded) practice for improving patient/population health. |
| Bucho-Gonzalez J, Herman PM, Larkey L, Menon U, Szalacha L. Startup and implementation costs of a colorectal cancer screening tailored navigation research study. Eval Prog Plan, 2021;85:101907. <https://doi.org/10.1016/j.evalprogplan.2021.101907> | Patients were referred to primary care which was not considered an evidence-based method under study. |
| Clarke CS, Vindrola-Padros C, Levermore C, Ramsay AIG, Black GB, Pritchard-Jones K, et al. How to cost the implementation of major system change for economic evaluations: case study using reconfigurations of specialist cancer surgery in part of London, England. Appl Health Econ Health Policy. 2021;19:797–810. https://doi.org/10.1007/s40258-021-00660-6 | A major system change was conducted, which cannot be considered an evidence-based (or empirically grounded) practice for improving patient/population health. Not an exclusive health focus. |
| Enggasser LJ, Livingston AN, Ameral V, Brief JD, Rubin A, Helmuth E, et al. Public implementation of a web-based program for veterans with risky alcohol use and PTSD: a RE-AIM evaluation of VetChange. J Subst Abuse Treat. 2021;122:108242. https://doi.org/10.1016/j.jsat.2020.108242. | No specific strategy to support implementation was used. Not conducted in a health care setting. |
| Isaacs AN, Dalziel K, Sutton K, Maybery D. Referral patterns and implementation costs of the Partners in Recovery initiative in Gippsland: learnings for the National Disability Insurance Scheme. Aust Psychiatry. 2018;26(6):586–9. <https://doi.org/10./1039856218759408> | No specific strategy was used. A referral system cannot be considered an evidence-based practice. |
| Kypridemos C, Collins B, McHale P, Bromley H, Parvulescu P, Capewell S, et al. Future cost-effectiveness and equity of the NHS Health Check cardiovascular disease prevention programme: Microsimulation modelling using data from Liverpool, UK. PLoS Med. 2018;15(5):e1002573. <https://doi.org/10.1371/journal.pmed.1002573> | Not conducted in a health care setting. |
| Kaufman BG, Holland DE, Vanderboom CE, Ingram C, Wild EM, Dose AM, et al. Implementation costs of technology-enhanced transitional palliative care for rural caregivers. Am J Hosp Palliat Care. 2024;41(1):38–44. <https://doi.org/10.1177/10499091231156145> | Health care workers as intended recipients. |
| Larson BA, Ngoma T, Silumbe K, Rutagwera MR, Hamainza B, Winters AM, et al. A framework for evaluating the costs of malaria elimination interventions: an application to reactive case detection in Southern Province of Zambia, 2014. Malar J. 2016 ;15(1):408. <https://doi.org/10.1186/s12936-016-1457-5> | Health care workers as intended recipients. |
| Meyer C, Golenko X, Cyarto EV, O'Keefe F, Bonney G, Min M, et al. Weaving Evidence into Action for Veterans with Dementia (WEAVE): evaluation of implementation into long-term care practice. J Eval Clin Pract. 2023;29(6):903–14. <https://doi.org/10.1111/jep.13852> | Not conducted in a health care setting. |
| Modi D, Somen S, Prakash V, Dave K. Anand A, Desai S, et al. Costing and cost-effectiveness of a mobile health intervention (ImTeCHO) in improving infant mortality in tribal areas of Gujarat, India: cluster randomized controlled trial. JMIR Mhealth Uhealth. 2020;8(10):e17066. <https://doi.org/10.2196/17066> | Not conducted in a health care setting. |
| van der Meer E, van Dongen J, Boot C, van der Gulden J, Bosmans J, Anema J. Economic evaluation of a multifaceted implementation strategy for the prevention of hand eczema among healthcare workers in comparison with a control group: the Hands4U study. Acta Derm Venereol. 2016;96(4):499–504. <https://doi.org/10.2340/00015555-2287> | Health care workers as intended recipients. |
| Shepard, D. , Shen, J. , Yant, B. , Denkmann, D. , Savage, P. , Collier, R. , Katz, B. , Ades, P. & Gaalema, D. (2025). Cost-Effectiveness of Case Management and Financial Incentives to Increase Participation in Cardiac Rehabilitation. *Journal of Cardiopulmonary Rehabilitation and Prevention, 45* (4), 278-285. doi: 10.1097/HCR.0000000000000952. | The implementation strategies were directed at patients. |
| Schouw D, Mash R, Ameh P, Fatusin BB, Engmann S. Evaluating the implementation of online research training and mentorship among early-career family physicians in sub-Saharan Africa. Prim Health Care Res Dev. 2025 Jan 9;26:e3. doi: 10.1017/S146342362400063X. PMID: 39781644; PMCID: PMC11735121. | Could no be considerate to be an evidence-based intervention |
| Federici C, Verykiou M, Cavazza M, Olomi W, Irakiza P, Kayumba K, Rodriguez E, Castillo Mendoza L, Malvolti S, Hugo C, Ntinginya N, Camara S, Sabi I, Sano N, Sibomana H, Condo J, Torbica A, Jommi C, Mantel C, Mangiaterra V. The Costs of Digital Health Interventions to Improve Immunization Data in Low- and Middle-Income Countries: Multicountry Mixed Methods Study doi: 10.2196/62746 | No implementation strategy |
| Haddad EN, Miles R, Alejandro-Rodriguez M, Gorenflo MP, Misirang A, Barbarotta S, Phillips W, Bharmal N, Yepes-Rios M. Feasibility of self-investment in a medically tailored meals program by a large health enterprise: Cleveland Clinic experience. Nutr Health. 2025 Dec;31(4):1503-1513. doi: 10.1177/02601060241307980. Epub 2025 Jan 24. PMID: 39849975. | No implementation strategy |

## Table S2. The Drummond checklist for assessing economic evaluation

| **Quality assessment item** | | **Barbosa et al. (2022)** | | **Barbosa et al. (2018)** | **Bartholomew et al. (2021)** | **Blackburn et al. (2025)** | **Chavez et al. (2023)** | **Christensen et al. (2026)** | **Duan et al. (2021)** | **Hoeft et al. (2019)** | **Jordan et al. (2019)** | **Keenan-Devlin (2024)** | **Kurzawa et al. (2021)** |
| --- | --- | --- | --- | --- | --- | --- | --- | --- | --- | --- | --- | --- | --- |
| **Study design** | |  | |  |  |  |  |  |  |  |  |  |  |
| The research question is stated. | | Yes | | Yes | Yes | Yes | Yes | Yes | Yes | Yes | Yes | Yes | Yes |
| The economic importance of the research question is stated. | | Yes | | Yes | Yes | Yes | Yes | Yes | Yes | Yes | Yes | Yes | Yes |
| The viewpoint(s) of the analysis are clearly stated and justified. | | Yes | | Yes | Yes | Yes | Yes | Yes | Yes | No | Yes | No | Yes |
| The rationale for choosing alternative programmes or interventions compared is stated. | | Yes | | Yes | Yes | Yes | Yes | Yes | Yes | No | No | No | Yes |
| The alternatives being compared are clearly described (enough detail to be able to relate the information on costs and outcomes to the alternative courses of action). | | Yes | | Yes | Yes | Yes | Yes | Yes | Yes | No | No | No | No |
| The form of economic evaluation used is stated. | | Yes | | Yes | Yes | Yes | Yes | Yes | Yes | Yes | Yes | Yes | Yes |
| The choice of form of economic evaluation is justified in relation to the questions addressed. | | Yes | | Yes | Yes | Yes | Yes | Yes | Yes | Yes | Yes | Yes | Yes |
| **Data collection** | |  | |  |  |  |  |  |  |  |  |  |  |
| The source(s) of effectiveness estimates used are stated. | | Yes | | No | No | No | No | Yes | Yes | No | No | No | Yes |
| Details of the design and results of effectiveness study are given (if based on a single study). | | Yes | | No | No | Yes | Yes | Yes | No | No | No | No | Yes |
| Details of the methods of synthesis or meta-analysis of estimates are given (if based on a synthesis of a number of effectiveness studies). | | NA | | NA | NA | NA | NA | NA | NA | NA | NA | NA | NA |
| The primary outcome measure(s) for the economic evaluation are clearly stated. | | Yes | | NA | NA | NA | NA | Yes | NA | NA | NA | NA | Yes |
| Methods to value benefits are stated. | | Yes | | NA | NA | NA | NA | NA | NA | NA | NA | NA | Yes |
| Details of the subjects from whom valuations were obtained were given | | Yes | | NA | NA | NA | NA | NA | NA | NA | NA | NA | Yes |
| Productivity changes (if included) are reported separately. | | No | | No | No | No | No | NA | NA | No | No | No | No |
| The relevance of productivity changes to the study question is discussed. | | No | | No | No | No | No | Yes | NA | No | No | No | No |
| Quantities of resource use are reported separately from their unit costs. | | Yes | | Yes | Yes | No | Yes | Yes | Yes | Yes | Yes | No | Yes |
| Methods for the estimation of quantities and unit costs are described. | | Yes | | Yes | Yes | Yes | Yes | Yes | Yes | Yes | Yes | No | Yes |
| Currency and price data are recorded. | | Yes | | Yes | Yes | Yes | Yes | Yes | Yes | Yes | Yes | Yes | Yes |
| Details of currency of price adjustments for inflation or currency conversion are given. | | Yes | | Yes | Yes | Yes | Yes | Yes | Yes | Yes | Yes | Yes | Yes |
| Details of any model used are given. | | NA | | NA | NA | NA | NA | NA | Yes | NA | NA | NA | NA |
| The choice of model used and the key parameters on which it is based are justified. | | NA | | NA | NA | NA | NA | NA | Yes | NA | NA | NA | NA |
|  |  | | **Analysis and interpretation of result** | | | | | | | | | | |
| Time horizon of costs and benefits is stated. | | Yes | | Yes | Yes | Yes | Yes | Yes | Yes | No | Yes | No | No |
| The discount rate(s) is stated. | | No | | NA | NA | NA | NA | No | No | NA | NA | NA | Yes |
| The choice of discount rate(s) is justified | | NA | | NA | NA | NA | NA | NA | NA | NA | NA | NA | Yes |
| An explanation is given if costs and benefits are not discounted. | | No | | No | No | No | No | No | No | No | No | No | NA |
| Details of statistical tests and confidence intervals are given for stochastic data | | Yes | | No | No | No | No | No | No | No | No | No | No |
| The approach to sensitivity analysis is given. | | Yes | | No | Yes | No | Yes | No | No | No | No | No | Yes |
| The choice of variables for sensitivity analysis is justified. | | Yes | | No | Yes | No | Yes | No | Yes | No | No | No | Yes |
| The ranges over which the variables are varied are justified. | | Yes | | No | Yes | No | Yes | No | Yes | No | No | No | Yes |
| Relevant alternatives are compared. | | Yes | | Yes | Yes | Yes | Yes | Yes | Yes | Yes | No | No | Yes |
| Incremental analysis is reported. | | Yes | | No | No | No | No | Yes | No | No | No | No | Yes |
| Major outcomes are presented in a disaggregated as well as aggregated form. | | Yes | | Yes | Yes | Yes | Yes | Yes | Yes | Yes | Yes | No | Yes |
| The answer to the study question is given. | | Yes | | Yes | Yes | Yes | Yes | Yes | Yes | Yes | Yes | Yes | Yes |
| Conclusions follow from the data reported. | | Yes | | Yes | Yes | Yes | Yes | Yes | Yes | Yes | Yes | Yes | Yes |
| Conclusions are accompanied by the appropriate caveats. | | Yes | | Yes | Yes | Yes | Yes | Yes | Yes | Yes | Yes | Yes | Yes |
| **Total score** | | 27 | | 17 | 20 | 17 | 21 | 21 | 22 | 13 | 14 | 10 | 26 |

|  | | **Lane et al. (2021)** | | **Lang et al. (2017)** | | **Nichols et al. (2020)** | | **Oxlade et al. (2021)** | **Parkinson et al. (2026)** | **Rahja et al. (2020)** | **Rose et al. (2025)** | **Wagner et al. (2021)** | **Willcox et al. (2019)** | **Woolf et al. (2024)** | **Yeung et al. (2020)** |
| --- | --- | --- | --- | --- | --- | --- | --- | --- | --- | --- | --- | --- | --- | --- | --- |
| **Study design** | |  | |  | |  | |  |  |  |  |  |  |  |  |
| The research question is stated. | | Yes | | Yes | | Yes | | Yes | Yes | Yes | Yes | Yes | Yes | Yes | **Yes** |
| The economic importance of the research question is stated. | | Yes | | Yes | | Yes | | Yes | Yes | Yes | Yes | Yes | Yes | Yes | Yes |
| The viewpoint(s) of the analysis are clearly stated and justified. | | Yes | | No | | No | | Yes | Yes | Yes | No | No | Yes | Yes | Yes |
| The rationale for choosing alternative programmes or interventions compared is stated. | | Yes | | Yes | | Yes | | Yes | Yes | Yes | Yes | Yes | No | Yes | Yes |
| The alternatives being compared are clearly described (enough detail to be able to relate the information on costs and outcomes to the alternative courses of action) | | No | | Yes | | Yes | | Yes | Yes | Yes | Yes | Yes | No | Yes | No |
| The form of economic evaluation used is stated. | | Yes | | Yes | | Yes | | Yes | Yes | Yes | Yes | Yes | Yes | Yes | Yes |
| The choice of form of economic evaluation is justified in relation to the questions addressed. | | Yes | | Yes | | Yes | | Yes | Yes | Yes | Yes | Yes | Yes | Yes | Yes |
| **Data collection** | |  | |  | |  | |  |  |  |  |  |  |  |  |
| The source(s) of effectiveness estimates used are stated. | | NA | | NA | | NA | | Yes | Yes | NA | NA | NA | NA | NA | NA |
| Details of the design and results of effectiveness study are given (if based on a single study). | | Yes | | NA | | Yes | | Yes | Yes | NA | Yes | Yes | NA | Yes | NA |
| Details of the methods of synthesis or meta-analysis of estimates are given (if based on a synthesis of a number of effectiveness studies). | | NA | | NA | | NA | | NA | NA | NA | Yes | NA | NA | NA | NA |
| The primary outcome measure(s) for the economic evaluation are clearly stated. | | NA | | NA | | NA | | Yes | Yes | Yes | Yes | NA | NA | NA | NA |
| Methods to value benefits are stated. | | NA | | NA | | NA | | Yes | Yes | Yes | No | NA | NA | NA | NA |
| Details of the subjects from whom valuations were obtained were given | | NA | | NA | | NA | | NA | NA | YES | NA | YES | NA | NA | NA |
| Productivity changes (if included) are reported separately. | | NA | | NA | | NA | | NA | NA | NA | No | NA | NA | NA | NA |
| The relevance of productivity changes to the study question is discussed. | | NA | | NA | | NA | | NA | NA | NA | Yes | NA | NA | NA | NA |
| Quantities of resource use are reported separately from their unit costs. | | No | | Yes | | Yes | | Yes | Yes | Yes | Yes | Yes | Yes | Yes | Yes |
| Methods for the estimation of quantities and unit costs are described. | | Yes | | Yes | | Yes | | Yes | Yes | Yes | Yes | Yes | Yes | Yes | Yes |
| Currency and price data are recorded. | | Yes | | Yes | | Yes | | Yes | Yes | Yes | Yes | Yes | Yes | Yes | Yes |
| Details of currency of price adjustments for inflation or currency conversion are given. | | Yes | | Yes | | Yes | | Yes | Yes | Yes | Yes | Yes | Yes | No | Yes |
| Details of any model used are given. | | NA | | NA | | NA | | Yes | Yes | NA | NA | NA | NA | NA | NA |
| The choice of model used and the key parameters on which it is based are justified. | | NA | | NA | | NA | | Yes | Yes | NA | NA | NA | NA | NA | NA |
|  |  | |  | |  | |  |  |  |  |  |  |  |  |  |
| Time horizon of costs and benefits is stated. | | Yes | | No | | No | | Yes | Yes | Yes | Yes | No | Yes | No | Yes |
| The discount rate(s) is stated. | | NA | | NA | | Yes | | No | Yes | Yes | No | NA | Yes | NA | NA |
| The choice of discount rate(s) is justified | | No | | No | | Yes | | NA | Yes | Yes | NA | No | Yes | No | No |
| An explanation is given if costs and benefits are not discounted. | | No | | No | | No | | No | NA | No | No | No | No | No | No |
| Details of statistical tests and confidence intervals are given for stochastic data | | No | | No | | No | | Yes | Yes | No | No | Yes | No | No | No |
| The approach to sensitivity analysis is given. | | Yes | | No | | Yes | | Yes | Yes | Yes | Yes | No | No | No | Yes |
| The choice of variables for sensitivity analysis is justified. | | Yes | | No | | Yes | | Yes | Yes | Yes | Yes | No | No | No | Yes |
| The ranges over which the variables are varied are justified. | | Yes | | No | | Yes | | Yes | Yes | Yes | Yes | Yes | No | No | Yes |
| Relevant alternatives are compared. | | Yes | | Yes | | Yes | | Yes | Yes | Yes | Yes | Yes | Yes | Yes | Yes |
| Incremental analysis is reported. | | No | | No | | Yes | | Yes | Yes | No | No | Yes | No | No | No |
| Major outcomes are presented in a disaggregated as well as aggregated form. | | No | | No | | Yes | | Yes | Yes | Yes | Yes | Yes | Yes | Yes | Yes |
| The answer to the study question is given. | | Yes | | Yes | | Yes | | Yes | Yes | Yes | Yes | Yes | Yes | Yes | Yes |
| Conclusions follow from the data reported. | | Yes | | Yes | | Yes | | Yes | Yes | Yes | Yes | Yes | Yes | Yes | Yes |
| Conclusions are accompanied by the appropriate caveats. | | Yes | | Yes | | Yes | | Yes | Yes | Yes | Yes | Yes | Yes | Yes | Yes |
| **Total score** | | 18 | | 14 | | 22 | | 28 | 30 | 25 | 22 | 21 | 17 | 16 | 19 |

NA, not appropriate.
